# Clinically accessible amplitude-based multiplex ddPCR assay for tryptase genotyping

**DOI:** 10.1038/s41598-024-52983-8

**Published:** 2024-01-29

**Authors:** Manca Svetina, Julij Šelb, Jonathan J. Lyons, Peter Korošec, Matija Rijavec

**Affiliations:** 1 University Clinic of Respiratory and Allergic Diseases Golnik, Golnik, Slovenia; 2 Biotechnical Faculty, University of Ljubljana, Ljubljana, Slovenia; 3 Faculty of Medicine, University of Ljubljana, Ljubljana, Slovenia; 4 National Institute of Allergy and Infectious Diseases, National Institutes of Health, Bethesda, USA; 5 Faculty of Pharmacy, University of Ljubljana, Ljubljana, Slovenia; 6 Faculty of Medicine, University of Maribor, Maribor, Slovenia

**Keywords:** DNA, Disease genetics, Genetic testing, Genetic predisposition to disease, Mast cells, Risk factors

## Abstract

Hereditary α tryptasemia (HαT) is an autosomal dominant trait characterized by increased *TPSAB1* copy number (CN) encoding α-tryptase. The determination of HαT is being discussed as an important biomarker to be included in risk assessment models and future diagnostic algorithms for patients with mastocytosis and anaphylaxis. Due to the complex genetic structure at the human tryptase locus, genetic testing for tryptase gene composition is presently notably limited and infrequently pursued. This study aimed to develop, optimise and validate a multiplex droplet digital PCR (ddPCR) assay that can reliably quantify α- and β-tryptase encoding sequences in a single reaction. To optimise the ddPCR conditions and establish an amplitude-based multiplex ddPCR assay, additional primers and probes, a thermal gradient with varying annealing temperatures, different primers/probe concentrations, and various initial DNA quantities were tested. Results obtained from all 114 samples analysed using multiplex ddPCR were identical to those obtained through the use of original duplex assays. Utilizing this multiplex ddPCR assay, in contrast to conducting distinct duplex ddPCRs, presents noteworthy benefits for tryptase genotyping. These advantages encompass a substantial threefold decrease in material costs and considerable time savings. Consequently, this approach exhibits high suitability and particularly captures interest for routine clinical implementation.

## Introduction

Among Western populations, there is a commonly observed genetic trait known as hereditary α-tryptasemia (HαT). This autosomal dominant condition is characterized by the presence of additional copies of the *TPSAB1* gene encoding α-tryptase, located on a single allele^1^. All known human mast cell tryptase genes are located in a compact cluster near the end of the short arm of chromosome 16. This cluster contains four paralogous genes, *TPSG1* (γ alleles), *TPSB2* (β2 and β3 alleles), *TPSAB1* (α and β1 alleles) and *TPSD1* (δ alleles). *TPSAB*1 and *TPSB2* sites harbour alleles of biologically relevant α- and β-tryptases, which are measured and reported as serum tryptase^2^. α-Tryptases exhibit minimal to no catalytic activity and are genetically absent in a significant portion of the human population. In contrast, β1- and β2-tryptases function as active peptidases. Limited information is available regarding β3-tryptases, although they are predicted to be active^3^. Increased copies of *TPSAB1* encoding α-tryptase are associated with elevated basal serum tryptase (BST) levels^4^. Whereas HαT is the common heritable cause for elevated BST, the clonal mast cell disorder systemic mastocytosis (SM) is an acquired condition that results in clinically rellevant BST elevations, and BST levels are used routinely as a biomarker to screen for this disorder^5–7^. Serum tryptase levels are also monitored to track disease progression and therapeutic response in SM. Therefore, when using BST levels as a minor diagnostic criterion for SM, adjustments to baseline tryptase should be considered^8,9^.

The consensus proposal and the 2022 World Health Organization classification have introduced new additions to the minor diagnostic criteria for SM, which now include an adjustment of BST levels in cases with HαT^10,11^. Additionally, the determination of HαT is currently being discussed as an important biomarker to be included in risk assessment models and future diagnostic algorithms for patients with mastocytosis. It remains to be shown whether mastocytosis patients with HαT benefit from more intense prophylactic management, or if HαT could even serve as a predictive biomarker for specific treatment approaches^8,9,12–16^.

Beyond clonal mast cell disease, increased BST levels are associated with adverse reactions to drugs, radiocontrast media and insect stings^17^. Even patients with slightly increased BST have a higher risk of anaphylaxis and experience more severe reactions^17–21^. Increased BST has also been identified as a moderate risk factor for side effects during venom immunotherapy^18,22^. While older studies did not determine the cause for elevated BST, multiple recent studies have confirmed that HαT is the primary cause for an association with elevated BST^9,14,23,24^. Thus, the relative risk for anaphylaxis is increased by either HαT or the presence of clonal mast cell disease, and the risks are additive^25^. Recent studies have demonstrated that individuals with HαT exhibit a greater propensity for experiencing more severe episodes of anaphylaxis^1,9,15,24,26^. Furthermore, tryptase gene composition, specifically the presence of α-tryptase encoding sequences, has been demonstrated as a potential biomarker for the severity of food allergy reactions^27^. Therefore, tryptase genotyping should be considered in the clinical evaluation of individuals with elevated BST and a history of, or at risk for, severe anaphylaxis^8,28,29^. The ability to stratify individuals based on risk and identify those predisposed to severe anaphylaxis is crucial, not only for the provision of anticipatory counseling, but also for guiding therapy^30,31^.

The accurate determination of the number of copies and isoforms present at the human tryptase locus has proven to be a technically challenging task, primarily due to the complex genetic structure, presence of multiple paralogs in a single locus, high density of GC-rich sequences, and significant sequence homology between α- and β-tryptases. Conventional next-generation sequencing, including exome or genome sequencing, Sanger sequencing or microarray technologies, have not been able to accurately delineate tryptase genotype or copy number (CN)^3,32,33^. To overcome this, an assay employing droplet digital polymerase chain reaction (ddPCR) technology, that accurately quantifies α- and β-tryptase CN arising from *TPSAB1* and *TPSB2* was developed by Lyons et al.^1^. ddPCR directly measures the quantity of a given DNA sequence relative to the quantity of a reference gene with conserved CN in the same reaction, allowing absolute quantification of α- and β-tryptase sequences and accurate determination of tryptase genotype^33^.

In addition to the standard detection of two targets using two different fluorophores, it is possible to increase the number of targets detected by varying parameters that affect PCR efficiency and end-point fluorescence. Increasing the number of potential targets per test in this manner can represent a significant improvement, dramatically augmenting the information output of each sample and saving on costs, required sample mass, and time. Implementing a multiplex ddPCR test for tryptase genotyping could also significantly reduce expenses associated with genetic testing for HαT, thereby facilitating its integration into routine clinical practice. Within this study, using primers and probes developed by Lyons et al.^1^ as well as additional sets of new primers and probes, we aimed to develop, optimise, and validate a multiplex ddPCR assay capable of reliably quantifying α- and β-tryptase encoding sequences in a single reaction.

## Methods

### Patients and samples

Development and validation of multiplex ddPCR was performed using samples collected from 111 patients with *Hymenoptera* venom-triggered anaphylaxis (HVA) and BST level of greater than or equal to 6.0 ng/mL, referred to the University Clinic Golnik. Samples underwent initial tryptase genotyping as part of a research collaboration between the University Clinic Golnik and the National Institutes of Health^34^. The cohort selection aimed to include various tryptase genotypes, as previous studies indicated a higher prevalence of HαT among individuals experiencing severe anaphylaxis^24,35^. Additionally, we extended the cohort by including three patients possessing exceedingly rare tryptase genotypes absent from the primary cohort (0α3β, 4α2β, 7α3β). This augmentation ensured the inclusion of nearly all reported tryptase genotypes to date, including the one with a high *TPSAB1* CN. Genomic DNA was extracted from 400 µL of EDTA-containing whole blood samples by using a QIAamp DNA Blood Mini Kit (Qiagen, Hilden, Germany) according to the manufacturer’s instructions. All patients had their BST levels routinely measured utilizing the commercially available fluorescence enzyme ImmunoCAP immunoassay (Thermo Fisher Scientific, Uppsala, Sweden). All individuals provided informed consent for the research protocol approved by the National Medical Ethics Committee of the Republic of Slovenia (KME 150/09/13). The study was carried out in accordance with the Declaration of Helsinki.

### Assay design

Custom primer/probe sets targeting α- and β-tryptases encoding sequences as reported were employed^1^. For the detection of reference CN invariant locus, additional primers and probes were designed with Primer3Plus^36^ and verified for sequence homology using the UCSC Genome Browser BLAT^37^. HPLC-purified probes dual labelled with either 5′-carboxyfluorescein (FAM) or 5′-hexachloro-fluorescein phosphoramidite (HEX) and 3′-BHQ1, as well as all MOPC purified primers, were synthesized by Macrogen Inc. (Seoul, South Korea). The accuracy of newly designed primers and probes was verified using a validated ddPCR Copy Number Reference Assay *AP3B1* (dHsaCP1000001) (Bio-Rad, Hercules, Calif, USA).

### Optimisation of the multiplex ddPCR assay and experimental workflow

To optimise the ddPCR conditions and establish an amplitude-based multiplex ddPCR assay, a thermal gradient with annealing temperature ranging from 58 to 64 °C, different primers (1350 to 2700 nM)/probe (250 to 750 nM) concentrations, and varying initial DNA quantities (10 ng, 25 ng, 55 ng) were tested. Following that, the multiplex ddPCR performance was compared with that of the original separate duplex ddPCRs using a commercialy available ddPCR Copy Number Reference Assay *AP3B1* (dHsaCP1000001) (Bio-Rad).

The reaction mix for all reactions, duplex as well as multiplex, was composed of 12.5 µL of 2× ddPCR Supermix for probes (No dUTP) (Bio-Rad), 1.25 µL of primer and probe mix, 5 µL of BamHI-treated genomic DNA and water to a final volume of 25 µL. A volume of 20 µL of the total mixture was used to generate droplets with the QX200 droplet generator (Bio-Rad) in an eight-channel DG8 cartridge and cartridge holder with 70 µL of Droplet Generation Oil for probes (Bio-Rad). 40 µL of the generated emulsion was transferred into a 96-well PCR plate (Bio-Rad), heat sealed with pierceable foil using PX1 PCR plate sealer (Bio-Rad), and amplified in a T100 Thermal Cycler (Bio-Rad)^38^. PCR amplification was performed with the following cycling parameters: initial denaturation at 95 °C for 10 min, followed by 40 cycles (at a temperature rate of 2.5 °C/s) of denaturation at 94 °C for 30 s and combined annealing/extension step at 60 °C for 1 min, and a final step at 98 °C for 10 min, ending at 4 °C. After amplification, the plate was directly transferred to the Droplet Reader, and the QX Manager software was used for data acquisition and data analysis.

## Results

### Various tryptase genotype samples

Previous tryptase genotyping^34^ revealed various tryptase genotypes, including those associated with HαT (Table 1). Consequently, this cohort, along with three additional samples possessing rare tryptase genotypes, provided an appropriate group for developing and validating the multiplex ddPCR assay.

**Table 1:** Tryptase genotypes of anaphylaxis patients^34^.

Genotype	Non HαT					HαT			
	2α2β	1α3β	0α4β	0α3β	1α2β	3α2β	2α3β	4α2β	7α3β
All patients, N	20	41	29	1	1	12	8	1	1

*HαT* hereditary α-tryptasemia.

### Primers and probes for invariant reference loci and CN reference assays

**Figure 1:**
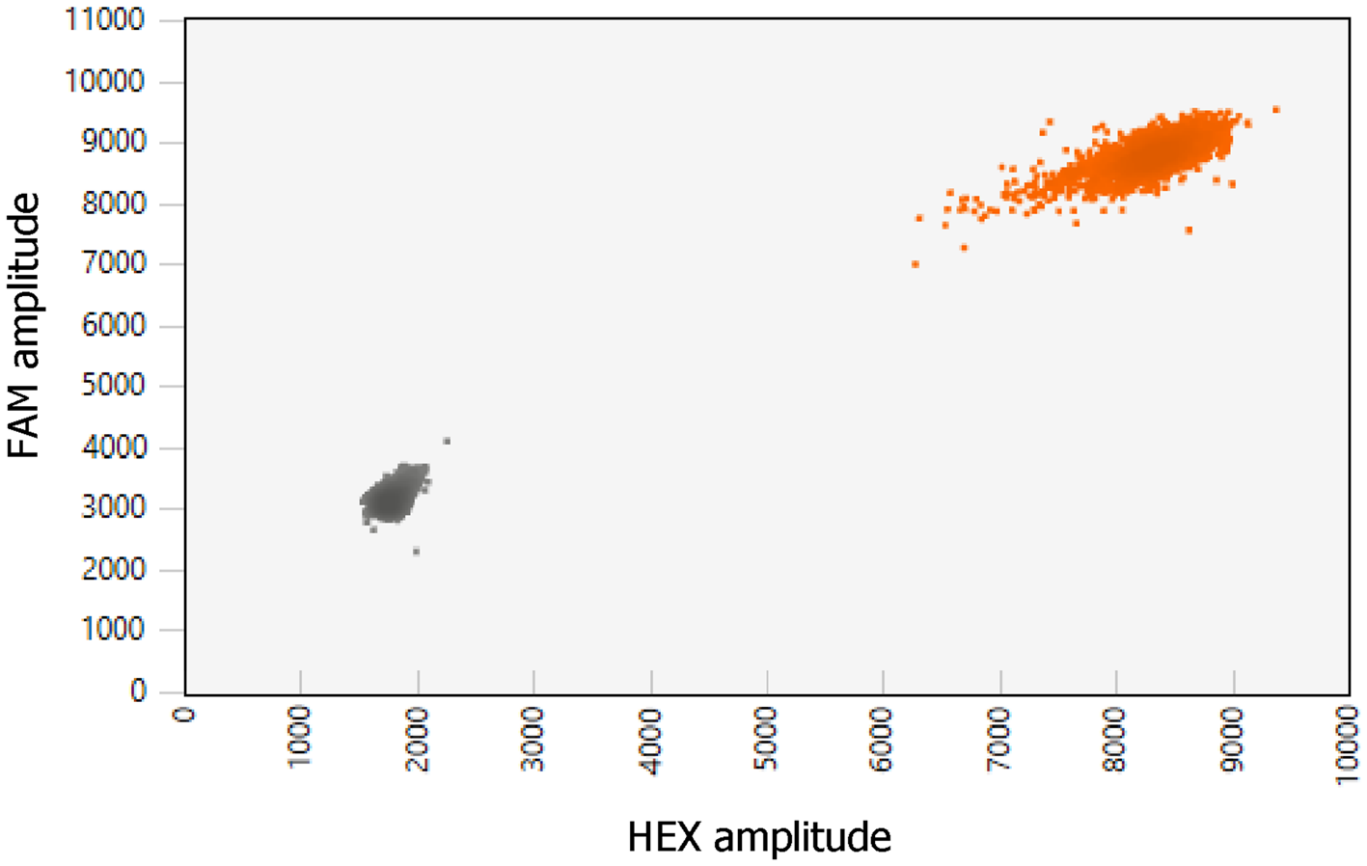
2D plot illustrates the specificity of the newly designed *AP3B1* CN reference assay. In the FAM channel, a validated ddPCR Copy Number Reference Assay *AP3B1* (Bio-Rad) was used, while the newly designed reference was analysed in the HEX channel. The plot displays a single cluster of positive droplets, indicating that the newly designed CN reference assay specifically detects the target of interest without generating non-specific signals. The assay also confirmed that the CN detected by the newly designed reference was consistent with the CN of the Bio-Rad reference.

Any region of the genome known or strongly suspected of maintaining an invariant CN can be utilized as a reference gene. Our study used two genes, *AP3B1* and *AGO1*, which had been suggested as suitable reference genes for copy number variation (CNV) analysis using ddPCR. *AGO1* (EIF2C1) (accession number NC_000001.11) is an argonaute RISC component 1 located on chromosome 1p34.3, and *AP3B1* (accession number NC_000005.10) is an adaptor-related protein complex 3 subunit β 1 located on chromosome 5p14.1. Both genes have two copies in diploid cells^39^. The primary objective of developing novel CN reference assays was to reduce costs in tryptase genotyping and provide an alternative to commercially available ddPCR Copy Number Reference Assay *AP3B1* (dHsaCP1000001) (Bio-Rad). In the process of designing primers and probes for the invariant reference loci *AP3B1* and *AGO1*, we adhered to several rules to ensure their optimal performance. Primers were designed with GC content ranging between 50 and 60%, and the melting temperature was targeted to fall within the range of 50 to 65 °C. The occurrence of longer repeated G and C bases was avoided, if possible Gs and Cs were placed at the 3' nucleotide of the primers. Forward and reverse primer sequences were examined to ensure the absence of 3′ complementarity, preventing the formation of primer dimers. The melting temperature of hydrolysis probes was set to be 3–10 °C higher than that of the corresponding primers. It was ensured that the probe length remained below 30 nucleotides, as the distance between the fluorophore and quencher influences the baseline signal intensity. The probe targeting α-tryptase was labelled with 5′-FAM, while the probes specific to β-tryptase and the CN reference loci were labelled with 5′-HEX. Primers and probes for target and reference loci are shown in Table 2. In a duplex ddPCR, utilizing both a newly designed *AP3B1* CN reference assay and a validated ddPCR Copy Number Reference Assay *AP3B1* (dHsaCP1000001) (Bio-Rad), we observed a single positive cluster of droplets (Fig. 1). This observation indicates the specificity and appropriateness of the designed CN reference assays for use as a CN reference. The *AGO1* CN reference assay was developed and validated at a later stage. This assay was specifically designed to offer an alternative method for determining CNVs in cases where the *AP3B1* CN reference assay is not applicable.

**Table 2:** Primer and probe sequences and their final concentrations used in multiplex ddPCR.

Target	Primer/probe	5′-sequence-3′	Final concentration [nmol/L]
*TPSAB1*, *TPSB2*	TPSAB1/TPSB2-F	TCCTGACCTGGCACCTGC	900
	TPSAB1/TPSB2-R	ACTCTCAGGCTCACCTGCCA	900
	TPSAB1 α probe	FAM-CTGCAGCAAGCGGGTATCGTC-BHQ1	250
	TPSAB1/TPSB2 β probe	HEX-CTGCAGCGAGTGGGCATCGT-BHQ1	250
*AP3B1*	AP3B1-F	CAGGTCTGCAGAGTCATA	1800
	AP3B1-R	CAGCCTCATCTCTCATACG	1800
	AP3B1 probe	HEX-AGCGGAATTGGAGAGGGAAGGTCAGCGA-BHQ1	400
*AGO1*	AGO1-F	TTCGGCTTTCACCAGTCT	1600
	AGO1-R	TCCCCACTCACCATCAAT	1600
	AGO1 probe	HEX-TGCGCCCTGCCATGTGGAAGATGA-BHQ1	350

### Optimisation of multiplex assay and optimal DNA quantity for *TPSAB1* CN assessment

To determine the optimal annealing temperature, we tested a range of temperatures above and below the calculated melting temperature of the primers. Among the tested temperatures, an annealing temperature 60 °C yielded the largest difference in fluorescence amplitude between positive and negative droplets (Details in Supplementary Fig. S1).

**Figure 2:**
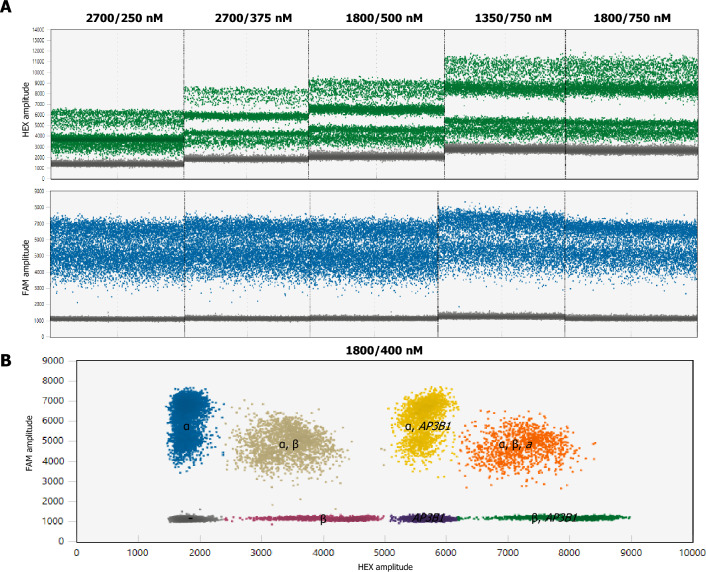
(**A**) Effect of primer/probe concentration on the fluorescence amplitude of droplets. By altering the concentrations of primers and probes specific to *AP3B1*, the amplitude is modified, subsequently affecting the positions of clusters. The primer and probe concentrations for α- and β-tryptase encoding sequences remain constant throughout experiments at 900 nM and 250 nM, respectively. (**B**) 2D plot generated using the optimised concentrations demonstrates the clear distinction of each target set within unique clusters.

The QX200 Droplet reader utilized in this study provides the capability to detect fluorescence signals in two distinct channels (FAM and VIC/HEX). However, an additional dimension of fluorescence amplitude can be exploited to enable the multiplexing of more than two assays simultaneously. The fundamental principle underlying multiplexing is to leverage variations in fluorescence amplitude signals to modify the spatial arrangement of droplet clusters within two-dimensional data plots. By varying the concentration of the TaqMan assay, it is possible to shift the end-point fluorescence amplitude. To achieve distinct segregation of clusters based on fluorescence amplitude, we had to optimise the concentrations of primers and probes. Our objective was to achieve the optimal segregation of clusters within one fluorescence channel, and based on the obtained results, we determined the most suitable final concentrations (Fig. 2, Table 2). These concentrations enable good separation of positive droplets among targets that employ probes labelled with the same fluorescent reporter. The multiplex assay comprises two sets of primers and three probes for the detection of three targets (α-tryptase, β-tryptase, *AP3B1*/*AGO1*). HEX channel is used to detect two targets (β-tryptase, *AP3B1*/*AGO1*).

**Figure 3:**
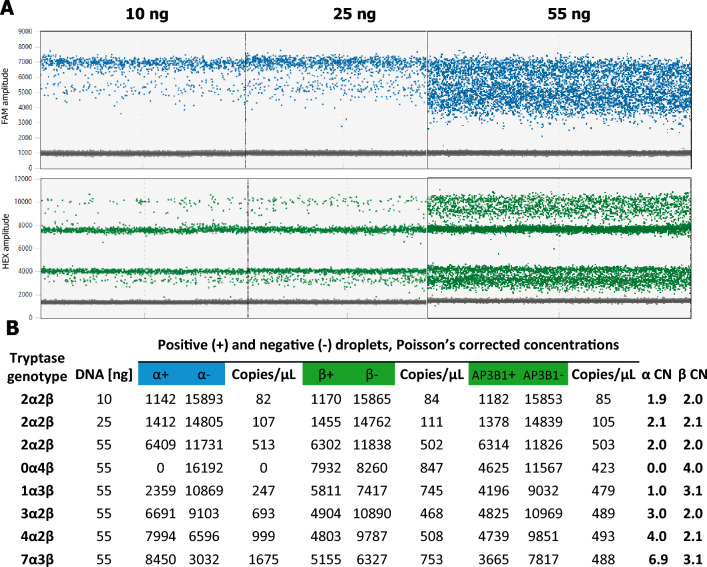
(**A**) The quantity of DNA has a direct impact on the percentage of positive and negative droplets observed. (**B**) When 55 ng of DNA is used, both the target and reference copies resides within the dynamic range of the instrument, ensuring accurate discrimination between consecutive CN states. CN was determined through the calculation of the ratio between the concentration of the target molecule and that of the reference molecule, multiplied by the number of copies of the reference gene present in the genome.

The primary technical challenge in CN assessment lies in the ability to discriminate between consecutive CN states. As the CN state increases, the percentage difference in target genomic material between states decreases. Discrimination between consecutive copy number states beyond CN 3 is determined by both, the concentration differences between consecutive CN states and the amount of DNA analysed per reaction well. The recommended dynamic range of the QX200 System spans from 1 to 120,000 copies per reaction. There are about 120,000 copies in 400 ng of human DNA, assuming 1 copy/haploid genome. Where a diploid target CN is expected to be 10 or less, approximately 0.2–1.0 reference gene copies per droplet (CPD) of DNA sample should be loaded per reaction well. Partitioning and subsampling errors are at their lowest point when approximately 1 CPD or 20,000 copies are used. This corresponds to 66 ng of human genomic DNA. At this point, negative droplets represent 37% of the total droplets^38^. We performed experiments employing distinct DNA concentrations (10 ng, 25 ng, 55 ng) across a range of *TPSAB1* CNs to ascertain the optimal initial DNA quantity. The DNA quantity of 55 ng was identified as optimal, as it ensured that both target and reference copies fell within the instrument’s dynamic range (Fig. 3).

### Comparison of multiplex ddPCR and duplex ddPCR tryptase genotyping

The performance of multiplex ddPCR was compared to original separate duplex ddPCRs. Genotyping of *TPSAB1* and *TPSB2* using duplex assays involved running two reaction wells: one targeting the α-tryptase sequence along with ddPCR Copy Number Reference Assay *AP3B1* (dHsaCP1000001) (Bio-Rad), and the other targeting the β-tryptase sequence along with ddPCR Copy Number Reference Assay *AP3B1* (dHsaCP1000001) (Bio-Rad). The initial multiplex ddPCRs were executed using the ddPCR Copy Number Reference Assay *AP3B1* (dHsaCP1000001) (Bio-Rad), followed by a subsequent multiplexing procedure employing the newly designed *AP3B1* CN reference assay in all included samples. The newly designed AGO1 CN reference assay underwent testing in a multiplex ddPCRs across 15 samples, incorporating all tryptase genotypes investigated in this study. The results obtained from all 114 samples analysed using multiplex ddPCR were found to be identical to those obtained through the use of original duplex assays (Supplementary Table S1). This demonstrates the comparable performance of the multiplex ddPCR approach in accurately determining CNVs. Utilizing this multiplex ddPCR with newly designed CN reference assays, in contrast to conducting two distinct duplex ddPCRs with ddPCR Copy Number Reference Assays *AP3B1* (dHsaCP1000001) (Bio-Rad), presents noteworthy benefits for determining *TPSAB1* CN. These advantages encompass a substantial threefold decrease in material costs and considerable time savings. Consequently, this approach exhibits high suitability and particularly captures interest for routine clinical implementation.

## Discussion

While the importance of HαT in diseases associated with mast cells cannot be understated^4,15,16,40^, its detection in clinical genetics laboratories remains limited^41^. The most robust and reliable method for accurately detecting CNVs in the *TPSAB1* and *TPSB2* genes is ddPCR^33^. While primarily utilized as a fundamental research tool, ddPCR is highly suitable for integration into clinical assays due to its exceptional precision and sensitivity. Notably, ddPCR is increasingly gaining prominence in the detection of specific categories of somatic genomic mutations, which hold prognostic significance in the context of various diseases^42^. Current ddPCR approaches for tryptase genotyping involve two reactions for each sample^1,23^, which can be relatively high in cost and time-consuming, potentially not meeting the requirements of clinically graded tests. In addition to the conventional ddPCR approach for single target detection within a channel, the capability exists to simultaneously detect multiple targets within the same channel. When developing multiplex ddPCR assays, two primary methodologies emerge: probe ratio-based multiplexing and amplitude-based multiplexing. In probe ratio-based multiplexing assays, certain targets are identified conventionally, while others are detected utilizing two probes conjugated with distinct dyes. In amplitude-based multiplex assay, targets are identified using probes conjugated with a single dye, albeit at varying concentrations^43^. Our study employed an amplitude-based strategy encompassing three distinct target sequences. The outcomes obtained from original independent duplex ddPCRs and multiplex ddPCR were found to be entirely consistent. All included tryptase genotypes were sucessfully determined, including the one with seven copies of α-tryptase encoding sequences. This indicates that the multiplex ddPCR assay would also enable the determination of *TPSAB1* CNs as high as 10, which represents the highest reported *TPSAB1* CN to date^7^.

An important consideration in the quantification of linked targets is that they undergo restriction digestion; otherwise, the assumption of a random and independent (Poisson) distribution of target molecules may not hold. Tandem repeat copies of the same target would co-localize in the same partition more frequently than expected by chance, leading to an underestimation of the true number of copies^43^. Therefore, the ddPCR for tryptase genotyping described herein must be executed on genomic DNA treated with the restriction endonuclease (BamHI), facilitating the accurate detection of multiple *TPSAB1* copies on a single allele. ddPCR-based tryptase genotyping assays rely on the assumption of normal CN of β-tryptase. Since deletions of β-tryptase encoding sequences have been documented to occur on rare occasions^44^, comparing CNV analysis on BamHI digested and undigested DNA, and examining family pedigrees along with Mendelian inheritance of tryptase haplotypes, can clarify seemingly discordant genotype or phenotype observations^1,44^. A similar strategy can be employed in discriminating between different HαT associated genotypes totalling four α-tryptase and two β-tryptase copies (4α2β), resulting in either αα/β:αα/β or α/β:ααα/β.

The implementation of multiplex ddPCR, coupled with the utilization of newly designed and validated primers and probes targeting CN invariant reference loci, results in a threefold reduction in material costs and substantial time savings for tryptase genotyping. This enhances the suitability of the diagnostic test for HαT and is expected to expand its accessibility in clinical settings. Recently, novel ddPCR systems have been introduced, incorporating additional channels to facilitate multiplexing. Within these systems, there is no requirement for probe ratio-based or amplitude-based multiplexing. Nevertheless, our described multiplex ddPCR for tryptase genotyping remains applicable without the need for introducing new probes. It is worth noting that the advanced ddPCR system, equipped with enhanced color detection capabilities, comes with a higher cost and is not anticipated to be readily available in clinical settings. At present, even the standard two-channel ddPCR system is not affordable in all institutions, but considering its diverse applications, we anticipate its evolution into a standard laboratory instrument in the near future.

Although none of the studied patients with HαT exhibited BST levels lower than 8.0 ng/mL, a BST level of greater than or equal to 8.0 ng/mL had a sensitivity of 100% (95% CI 84–100%), specificity of 71% (95% CI 61–79%), positive predictive value of 43%, and negative predictive value of 100% for the identification of patients with HαT. However, this calculation may be subject to potential bias, as tryptase genotyping was exclusively conducted in HVA patients exhibiting BST levels above 6 ng/mL. Neverheless, to ensure an accurate diagnosis of HαT, ddPCR is indispensable, as relying solely on BST measurements is inadequate^14^. Furthermore, the presence of elevated BST levels, after excluding a diagnosis of HaT, may identify acquired etiologies responsible for BST elevation, with the most prevalent etiologies being clonal myeloid disorders and renal insufficiency^6^. Moreover, recent research has demonstrated that the significance extends beyond the presence of HαT; the quantity of α-tryptases also emerges as a crucial factor^27,45^. Consequently, it is not only the presence of HαT that is important, but also the complete genotypic characterization of tryptase.

In conclusion, genetic testing for HαT, despite its demonstrated significance in diseases associated with mast cells, remains notably limited and infrequently conducted^7,15,16,35,41^. In response to current test limitations, we have developed a precise and robust multiplex ddPCR assay for the absolute quantification of α- and β-tryptase encoding sequences and, consequently, the accurate determination of tryptase genotype. The attributes of cost-effectiveness and rapid turnaround inherent to this methodology underscore its potential for facilitating clinical translation. Our findings suggest that the described multiplex ddPCR approach possesses the essential characteristics required to advance the application of genetic testing for HαT in clinical settings.

### Supplementary Information

Supplementary Information.

## Data Availability

The datasets generated during and/or analysed during the current study are available from the corresponding author on request.
