# Supplementary material for: Clinically accessible amplitude-based multiplex ddPCR assay for tryptase genotyping
Source: Sci Rep. 2024 Jan 29;14:2416. doi: 10.1038/s41598-024-52983-8 (PMC10825142; doi:10.1038/s41598-024-52983-8)
Supplement: Supplementary file 1 — Supplementary Information. [file 41598_2024_52983_MOESM1_ESM.pdf]

Clinically accessible amplitude-based multiplex ddPCR assay for tryptase genotyping

Manca Svetina[1,2], Julij Šelb[1,3], Jonathan J. Lyons[4], Peter Korošec[1,5,6], Matija Rijavec[1,2*]

[1]University Clinic of Respiratory and Allergic Diseases Golnik, Golnik, Slovenia

[2]Biotechnical Faculty, University of Ljubljana, Ljubljana, Slovenia

[3]Faculty of Medicine, University of Ljubljana, Ljubljana, Slovenia

[4]National Institute of Allergy and Infectious Diseases, National Institutes of Health, Bethesda

[5]Faculty of Pharmacy, University of Ljubljana, Ljubljana, Slovenia

[6]Faculty of Medicine, University of Maribor, Maribor, Slovenia

*Corresponding author: matija.rijavec@klinika-golnik.si

Supplemental data

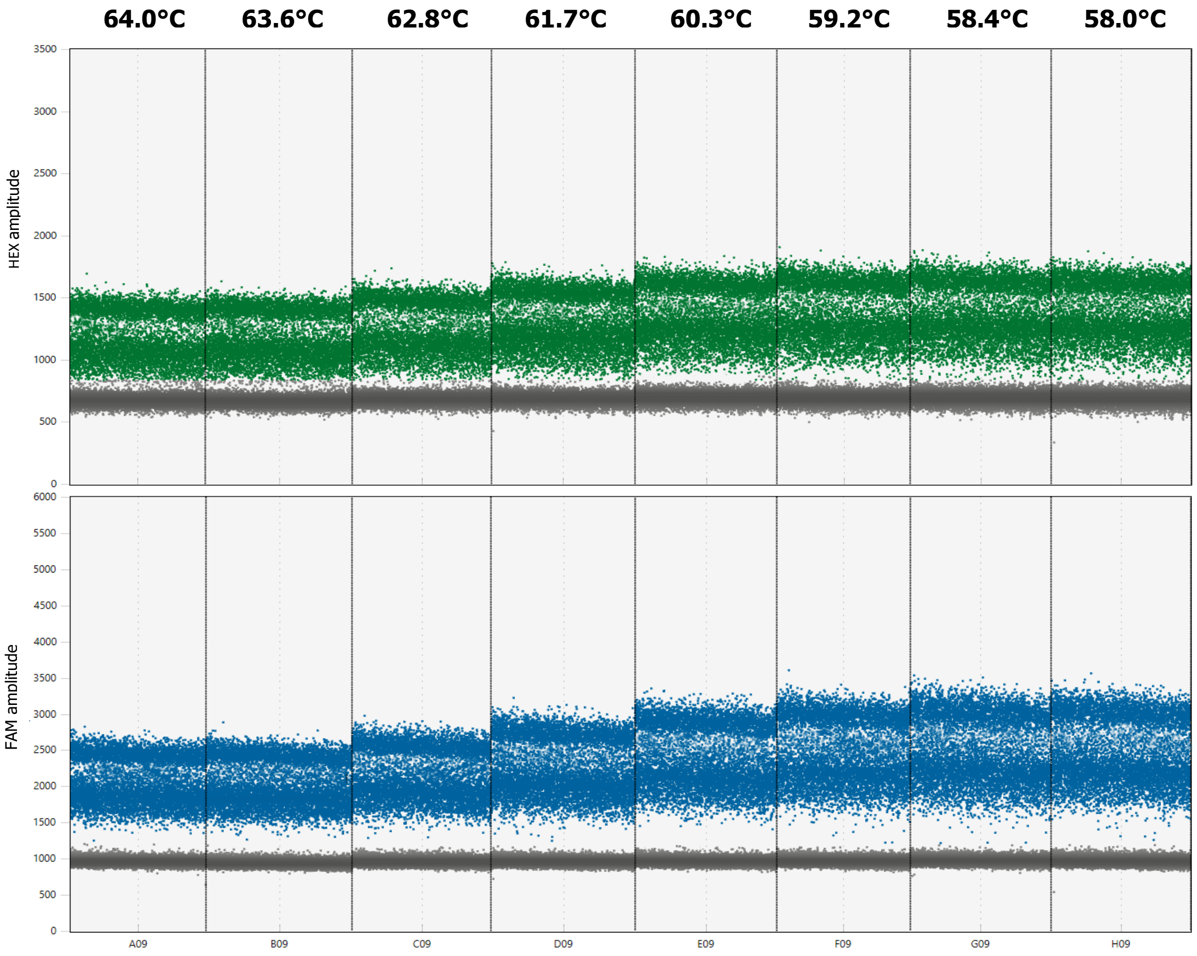

Supplementary Figure S1. Thermal gradient optimisation: To find the optimal annealing temperature, we tested a range of temperatures above and below the calculated melting temperature of the primers specific for α- and β-tryptase sequences. In order to ascertain a temperature for the proficient performance of both assays in the FAM channel, α-tryptase was analysed, while β-tryptase was analysed in the HEX channel. The largest fluorescence difference between the positive and negative droplets was determined at 60°C.

Supplementary Table S1. Obtained results for α- and β-tryptase copy numbers (CN) from original separate duplex ddPCRs and multiplex ddPCR utilizing distinct CN reference assays.

	Original separate duplex ddPCRs		Multiplex ddPCR (*AP3B1* CN reference assay (Bio-Rad))		Multiplex ddPCR (newly designed *AP3B1* CN reference assay)		Multiplex ddPCR (newly designed *AGO1* CN reference assay)	
	α	β	α	β	α	β	α	β
Patient 1	1	3	1	3	1	3	-	-
Patient 2	1	3	1	3	1	3	-	-
Patient 3	2	2	2	2	2	2	-	-
Patient 4	2	3	2	3	2	3	-	-
Patient 5	0	4	0	4	0	4	-	-
Patient 6	0	4	0	4	0	4	0	4
Patient 7	1	3	1	3	1	3	-	-
Patient 8	0	4	0	4	0	4	-	-
Patient 9	1	3	1	3	1	3	-	-
Patient 10	0	4	0	4	0	4	-	-
Patient 11	1	3	1	3	1	3	-	-
Patient 12	1	3	1	3	1	3	-	-
Patient 13	2	2	2	2	2	2	-	-
Patient 14	0	4	0	4	0	4	-	-
Patient 15	2	3	2	3	2	3	-	-
Patient 16	0	4	0	4	0	4	-	-
Patient 17	2	3	2	3	2	3	-	-
Patient 18	0	4	0	4	0	4	-	-
Patient 19	1	3	1	3	1	3	-	-
Patient 20	0	4	0	4	0	4	-	-
Patient 21	1	3	1	3	1	3	-	-
Patient 22	2	3	2	3	2	3	-	-
Patient 23	1	3	1	3	1	3	-	-
Patient 24	1	3	1	3	1	3	-	-
Patient 25	2	2	2	2	2	2	-	-
Patient 26	2	2	2	2	2	2	-	-
Patient 27	2	2	2	2	2	2	-	-
Patient 28	0	4	0	4	0	4	-	-
Patient 29	1	3	1	3	1	3	-	-
Patient 30	1	3	1	3	1	3	-	-
Patient 31	2	2	2	2	2	2	2	2
Patient 32	0	4	0	4	0	4	-	-
Patient 33	1	3	1	3	1	3	-	-
Patient 34	0	4	0	4	0	4	-	-
Patient 35	0	4	0	4	0	4	0	4
Patient 36	3	2	3	2	3	2	-	-
Patient 37	1	3	1	3	1	3	-	-
Patient 38	2	3	2	3	2	3	2	3
Patient 39	1	3	1	3	1	3	-	-
Patient 40	3	2	3	2	3	2	-	-
Patient 41	2	3	2	3	2	3	-	-
Patient 42	0	4	0	4	0	4	-	-
Patient 43	1	3	1	3	1	3	-	-
Patient 44	1	3	1	3	1	3	-	-
Patient 45	1	3	1	3	1	3	-	-
Patient 46	1	3	1	3	1	3	-	-
Patient 47	1	3	1	3	1	3	-	-
Patient 48	3	2	3	2	3	2	-	-
Patient 49	0	4	0	4	0	4	-	-
Patient 50	2	2	2	2	2	2	-	-
Patient 51	3	2	3	2	3	2	-	-
Patient 52	3	2	3	2	3	2	-	-
Patient 53	1	3	1	3	1	3	-	-

Patient 54	1	3	1	3	1	3	-	-
Patient 55	2	2	2	2	2	2	-	-
Patient 56	1	3	1	3	1	3	-	-
Patient 57	1	3	1	3	1	3	-	-
Patient 58	3	2	3	2	3	2	-	-
Patient 59	0	4	0	4	0	4	-	-
Patient 60	2	2	2	2	2	2	-	-
Patient 61	3	2	3	2	3	2	3	2
Patient 62	0	4	0	4	0	4	-	-
Patient 63	2	2	2	2	2	2	-	-
Patient 64	2	2	2	2	2	2	-	-
Patient 65	1	3	1	3	1	3	-	-
Patient 66	1	3	1	3	1	3	-	-
Patient 67	3	2	3	2	3	2	-	-
Patient 68	0	4	0	4	0	4	-	-
Patient 69	0	4	0	4	0	4	-	-
Patient 70	1	3	1	3	1	3	-	-
Patient 71	1	3	1	3	1	3	-	-
Patient 72	1	3	1	3	1	3	-	-
Patient 73	1	3	1	3	1	3	1	3
Patient 74	2	2	2	2	2	2	-	-
Patient 75	2	3	2	3	2	3	2	3
Patient 76	3	2	3	2	3	2	3	2
Patient 77	3	2	3	2	3	2	-	-
Patient 78	0	4	0	4	0	4	-	-
Patient 79	1	3	1	3	1	3	-	-
Patient 80	3	2	3	2	3	2	-	-
Patient 81	1	2	1	2	1	2	1	2
Patient 82	1	3	1	3	1	3	-	-
Patient 83	2	2	2	2	2	2	-	-
Patient 84	1	3	1	3	1	3	-	-
Patient 85	1	3	1	3	1	3	-	-
Patient 86	2	2	2	2	2	2	2	2
Patient 87	1	3	1	3	1	3	-	-
Patient 88	0	4	0	4	0	4	-	-
Patient 89	2	2	2	2	2	2	-	-
Patient 90	1	3	1	3	1	3	1	3
Patient 91	1	3	1	3	1	3	-	-
Patient 92	1	3	1	3	1	3	-	-
Patient 93	1	3	1	3	1	3	-	-
Patient 94	1	3	1	3	1	3	-	-
Patient 95	0	4	0	4	0	4	-	-
Patient 96	0	4	0	4	0	4	-	-
Patient 97	0	4	0	4	0	4	-	-
Patient 98	0	4	0	4	0	4	-	-
Patient 99	3	2	3	2	3	2	3	2
Patient 100	0	4	0	4	0	4	-	-
Patient 101	2	2	2	2	2	2	-	-
Patient 102	0	4	0	4	0	4	-	-
Patient 103	2	3	2	3	2	3	-	-
Patient 104	1	3	1	3	1	3	-	-
Patient 105	0	4	0	4	0	4	-	-
Patient 106	0	4	0	4	0	4	-	-
Patient 107	2	2	2	2	2	2	-	-
Patient 108	2	2	2	2	2	2	-	-
Patient 109	2	2	2	2	2	2	-	-
Patient 110	2	2	2	2	2	2	-	-
Patient 111	0	4	0	4	0	4	-	-
Patient 112*	0	3	0	3	0	3	0	3
Patient 113*	4	2	4	2	4	2	4	2
Patient 114*	7	3	7	3	7	3	7	3

*Additionaly included samples with rare tryptase genotypes
